# A Novel Workflow to Estimate Limb Orientation from Wearable Sensors to Monitor Infant Motor Development

**DOI:** 10.3390/s26072274

**Published:** 2026-04-07

**Authors:** David Song, William J. Kaiser, Sitaram Vangala, Rujuta B. Wilson

**Affiliations:** 1 Department of Psychiatry, University of California, Los Angeles, CA 90095, USA; davidsongxyz@ucla.edu; 2 Semel Institute for Neuroscience and Human Behavior, Los Angeles, CA 90095, USA; 3 Department of Electrical Engineering, University of California, Los Angeles, CA 90095, USA; kaiser@ee.ucla.edu; 4 Department of Medicine Statistics Core, David Geffen School of Medicine, University of California, Los Angeles, CA 90024, USA; svangala@mednet.ucla.edu

**Keywords:** infant, motor development, wearable sensors, orientation

## Abstract

Background: Wearable sensors have gained increasing popularity as an objective method for remotely monitoring infant movement in naturalistic settings. Over the first year of life, infants generate a wide range of motions, from goal-directed to spontaneous movement. These include linear movements, such as kicks, and orientation changes, such as postural transitions. Many sensor processing pipelines emphasize capturing linear movements through movement-generated acceleration while focusing less on information about orientation embedded in the gravitational part of the data. Here, we introduce a complementary gravity-referenced approach that extracts the gravitational component of accelerometer signals to estimate limb orientation, extending the reliable quantification of rich and detailed aspects of infant movement. Infant orientation has demonstrated clinical relevance, including associations with later neuromotor outcomes, and it can be used to chart infant motor development, motivating the development of objective methods to quantify orientation from sensor data. Methods: Wearable sensors (Opal APDM) were used to longitudinally evaluate infant motor activity recorded in sessions conducted at 3, 6, 9, and 12 months of age. We extracted data from a 5 min segment that has simultaneous video recordings. From these datasets, applying the gravity-referenced method, we computed pitch, roll, and yaw, angles that collectively describe limb orientation. We then quantified orientation variability using axis-specific circular standard deviations (SDs) for pitch, roll, and yaw and a multi-axis composite measure based on generalized variance. Results: Axis-specific circular SDs for pitch, roll, and yaw, as well as the composite generalized variance, increased significantly from 3 to 12 months (*p* ≤ 0.01 for each metric). Composite variability was strongly associated with Mullen gross motor outcomes at 9 and 12 months of age (r = 0.55, *p* < 0.001). Conclusions: Overall, gravity-referenced pitch, roll, and yaw provide rich orientation features that increased as infants develop more postural transitions. Furthermore, the orientation features correlated with standardized measures of infant motor function. These orientation metrics can complement traditional linear kinematic measures and improve our ability to granularly track infant motor development in the first year of life.

## 1. Introduction

Wearable technology has become a widely used method of measuring human movement. Commercial devices such as smart watches, foot pods and fitness trackers can quantify physical activity levels through step counts, gait speed, stride length, and distance traveled [1,2]. Research-grade devices with more advanced systems, such as inertial measurement units (IMUs), are capable of evaluating more detailed aspects of movements. This includes movement quantity, intensity, variability and coordination. Moreover, the versatility of wearable technology allows for monitoring in the home, lab, and other settings and across multiple parts of the body, including the limbs, waist, and trunk [3,4,5]. Wearable sensors can also overcome the limitations of standardized motor assessments, which can be time-intensive, require specialized training, and are often conducted in the laboratory setting [6,7]. As such, wearable sensors have become important tools for efficacy and feasibility studies of physical therapy and other motor interventions (e.g., CIMT and stroke rehabilitation) and monitoring a wide range of conditions such as Parkinson’s disease and multiple sclerosis [8,9,10,11], and a wide range of age groups from infant to geriatric populations.

In infant populations, wearable sensors have been utilized to evaluate infants with neurotypical development and those with a higher likelihood of developmental delays or genetic conditions. This includes infants with a high likelihood of autism (infants with an older sibling with autism), infants that have been in the neonatal intensive care unit (NICU), and infants with Down Syndrome, as well as infants with other more general developmental delays [12,13,14,15,16,17]. The high-frequency sampling of movement data acquired by the wearable sensor is particularly useful in assessing detailed aspects of infant movements. These data can be used to construct sensitive motor markers that can aid in developmental monitoring in infancy and early childhood and potentially provide early predictive markers of later developmental outcomes [18]. Studies of infant movements using wearables sensors have included measurements of arm and leg movements, as well as evaluation of postural control and developmental milestones. Here, we direct particular focus toward studies evaluating arm and leg movements, which often examine spontaneous movements in early infancy to goal-directed movements in later infancy. These studies have developed different sensor-based movement metrics to quantify infant movements, including movement intensity, variability, asymmetry, and complexity [16,17,19,20]. Indeed, this rich collection of metrics has supported numerous findings that highlight their value in medical applications involving early motor development. Our own team has developed wearable sensor-based measures of movement variability which can be used to predict ASD outcomes in infants and toddlers at elevated likelihood of autism as early as 12 and 18 months of age, respectively [16,17]. Low-intensity movements and increased activity during the night have also demonstrated an association with delays in motor milestones for infants with Down Syndrome [21].

The acceleration data, measured by an accelerometer, includes three contributions to acceleration: movement-related acceleration, gravitational acceleration, and acceleration measurement noise [22]. In studies similar to the ones described above, researchers commonly focus on movement-related acceleration to extract spontaneous arm and leg movements of infants from accelerometer data, supplementing their process with gyroscope and magnetometer data [23,24]. As a result, the gravitational component of accelerometer data has commonly been regarded as background information that interferes with the extraction of individual movements. Many studies aim to remove the gravitational component through a series of preprocessing steps such as frequency-based attenuation or direct subtraction. One strategy is to attenuate frequencies below or within a range of frequencies, since gravitational acceleration often appears as a low-frequency signal [25,26,27]. Another tactic is to subtract 9.81 m/s^2^, the magnitude of gravitational acceleration, from the overall magnitude of acceleration measured by the accelerometer [28,29,30]. The processing methods used in these studies are typically best suited for extracting linear kinematic metrics like overall activity level.

Although most analyses focus on linear movement acceleration, evaluation of the gravitational acceleration produced by the sensor can yield information about the detailed, multiaxis orientation of infant limbs and can identify important aspects of motor function, such as limb posture and coordination, that cannot be derived from movement-related acceleration alone. Additionally, since gravitational acceleration dominates the accelerometer signal in magnitude, most of the variation in accelerometer data reflects changes in sensor orientation. This allows gravity-based orientation features to be more stably estimated and interpretable than movement-related acceleration, particularly in infant recordings where movement signals are brief, irregular, and variable. In contrast, extracting movement-related acceleration requires separating dynamic motion from gravity, a process that is sensitive to residual noise and other estimation errors, especially when the remaining movement signal is small or sparse [31].

Typical patterns of limb orientation are crucial for the development of motor coordination, range of motion, and balance. Previous research has demonstrated that observable postural patterns in infants during everyday movements are associated with later neuromotor outcomes such as cerebral palsy [32]. Sensors and video imaging methods offer a detailed view of orientation, providing additional value in developing metrics to identify atypical motor development. Thus, orientation analysis of limbs through wearable sensors represents a promising avenue for enhancing the interpretability of infant movement in clinical research.

In this paper, we describe methods that can be used to leverage the gravitational component of wearable sensor data to study infant movement focusing on 3D orientation dynamics. We also demonstrate their potential for capturing additional sets of kinematic features that complement existing metrics and providing a continuous picture of infant movement. First, we provide background for the conventional orientation metrics pitch, roll, and yaw. Next, we describe the process we used to extract these orientation metrics from sensor data and introduce measures that can be derived from pitch, roll, and yaw. Finally, we will compute descriptive statistics for pitch, roll, and yaw in our infant sample to analyze their patterns of change over the first year of life and examine their association with standardized gross motor assessments.

## 2. Background

### 2.1. Pitch, Roll, and Yaw

**Figure 1:**
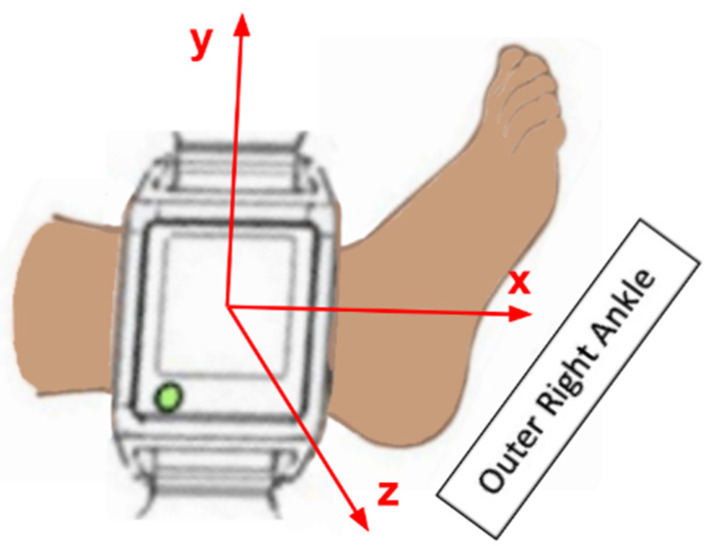
Sensor placement and coordinate frame (x, y, z) on an infant limb.

The orientation of our sensor in 3D space can be characterized by a sequence of rotations: yaw (the angle of rotation about the *z*-axis), pitch (the angle of rotation about the *y*-axis), and roll (the angle of rotation about the *x*-axis) (Figure 1).

As noted previously, accelerometer data can be decomposed into movement-related acceleration and gravitational acceleration. For orientation estimation, we utilize an alternative processing method of raw accelerometer data to isolate the gravitational acceleration instead of the typical movement-related acceleration. As a result, the isolated acceleration vector will represent the gravity vector (although we refer to this as the “gravity vector” for convenience, the accelerometer detects gravity by measuring the surface normal vector, which points opposite to gravitational acceleration).

**Figure 2:**
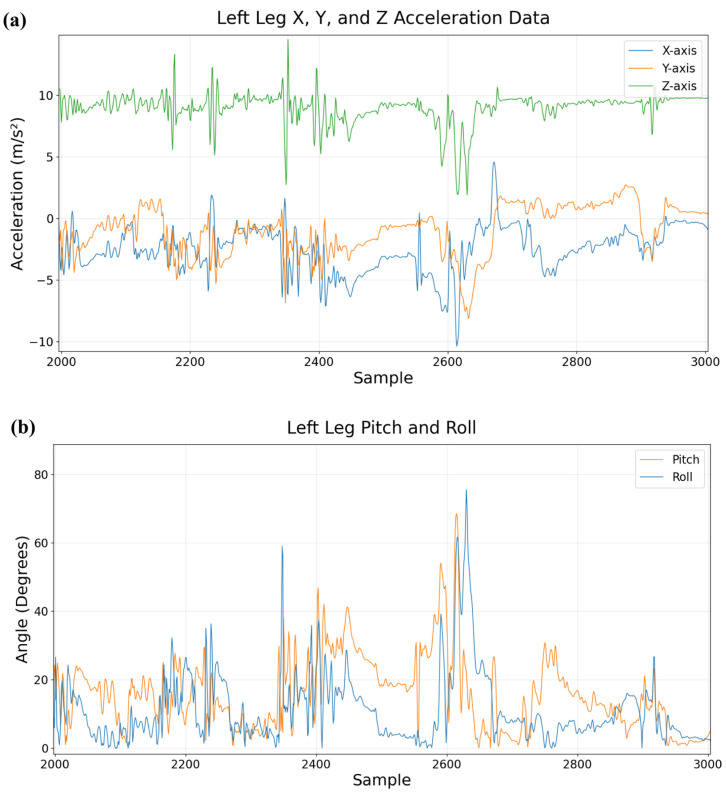
(**a**) Left leg tri-axial accelerometer data, (**b**) pitch and roll angles derived from accelerometer gravity vector (example segment), showing concordance between the raw accelerometer signal and the estimated angles.

Each 3D orientation of the sensor yields a unique orientation of the gravity vector in the sensor’s coordinate frame. For example, a sensor laid flat on a surface will produce a gravity vector with a +9.81 m/s^2^ component on the *z*-axis. If the sensor is rotated by 45° about the local *y*-axis (i.e., the sensor rotates relative to the world), the gravity vector will instead have a z-component of +9.81cos(45°) m/s^2^ and an x-component of +9.81sin(45°) m/s^2^. Using components of the gravity vector, we can extract pitch and roll with the following formulas (Figure 2) [33]:

$$Pitch=arctan\left(-\frac{X}{\sqrt{Y^{2}+Z^{2}}}\right)$$

$$Roll=arctan(\frac{Y}{Z})$$

**Figure 3:**
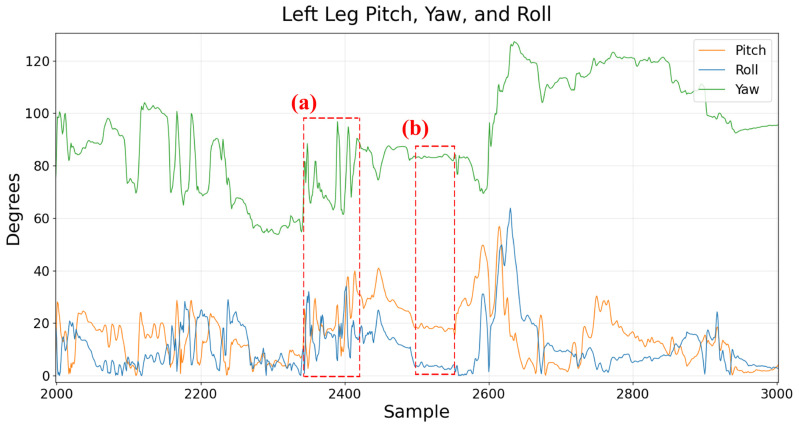
Pitch, yaw, and roll angles estimated using the Madgwick Filter. Region (**a**) indicates a bout of movement, while region (**b**) indicates a quiescent period.

To fully represent the 3D orientation at each frame, we must also include yaw. However, as is well known in inertial sensing development, yaw cannot be computed from accelerometer data alone (See Appendix A.1). Instead, we can apply methods based on the development of a sensor data fusion algorithm combining acceleration (from accelerometer sensors), rotation rate (from rate gyroscope sensors), and Earth’s magnetic field (from magnetometer sensors) [34]. An implementation of these principles, referred to as the Madgwick filter, has been developed and validated for applications in inertial sensing for pitch, roll, and yaw measurement. Results fusing accelerometer, gyroscope, and magnetometer signals to compute the pitch, roll, and yaw angles are shown in Figure 3.

**Figure 4:**
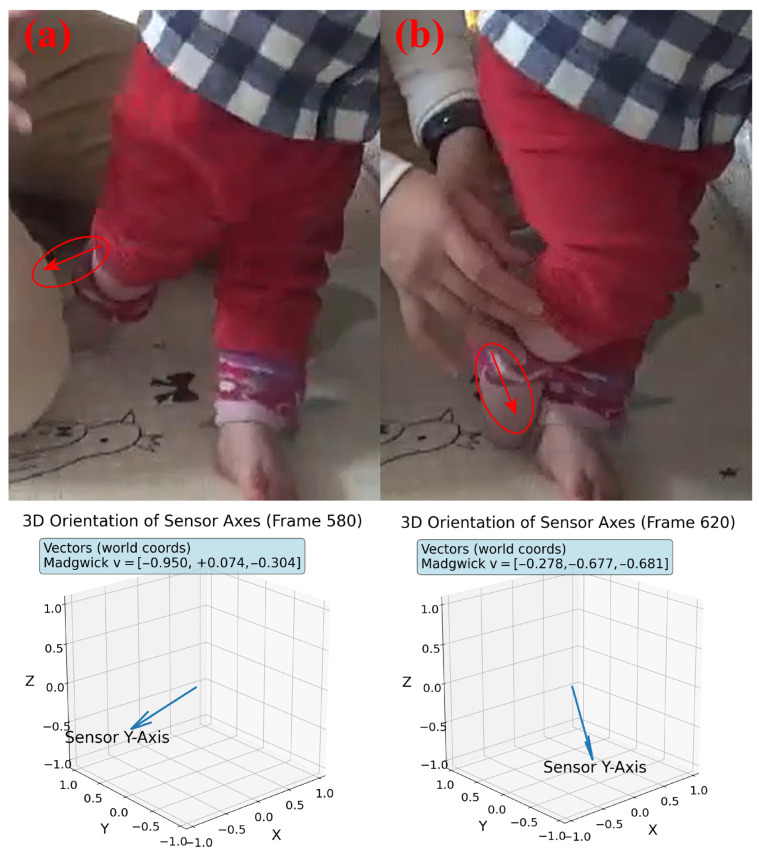
Example of 3D orientation tracking using the pitch, roll, and yaw angles produced by the Madgwick filter. (**a**,**b**) represent two different orientations of the infant’s right leg. The first row represents frames selected from a video recording of the infant with red arrows indicating the orientation of the sensor’s *Y*-axis. The second row represents our calculated orientation of the sensor’s *Y*-axis.

Using pitch, roll, and yaw calculated by the Madgwick filter, we reconstruct 3D orientation vectors to represent the orientation of our sensors. We then compare these vectors to the sensor orientation from a 5 min baseline recording of infant motion (Figure 4).

In some research and practical contexts, sensors may lack gyroscope or magnetometer inputs [17,19]. In such cases, our stream of information is limited to pitch and roll, which only require accelerometer data. Thus, we decided to evaluate the effectiveness of pitch and roll alone in calculating coarse metrics such as composite angular velocity in our context of infant limb movement.

**Figure 5:**
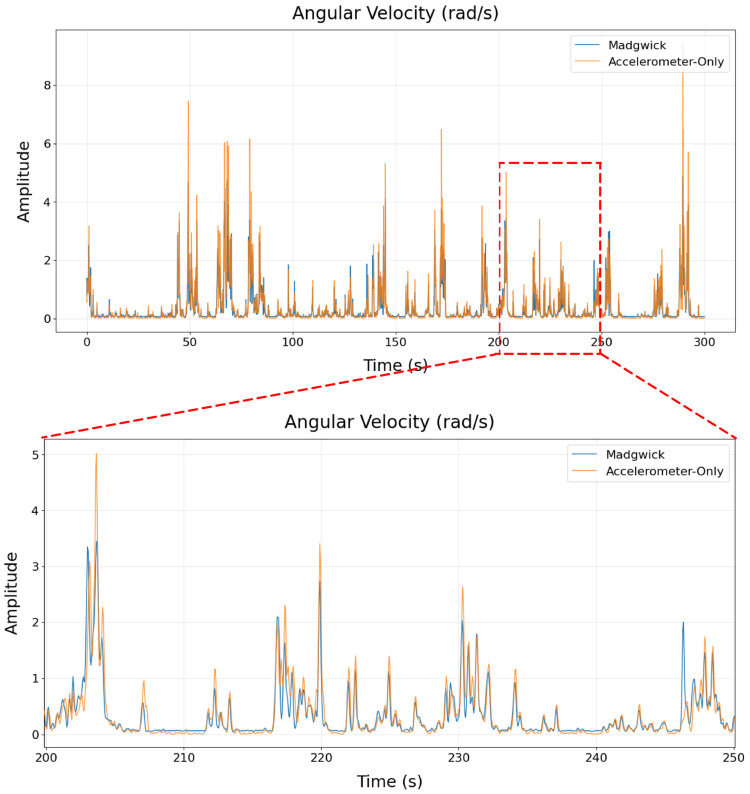
Comparison of Madgwick-filtered and accelerometer-only angular velocity estimates on example data (zoomed interval shown).

Using our definitions of pitch, roll, and yaw, we computed a time series for each angle across the sensor recording. We then constructed a composite angular velocity time series (See Appendix A.2), using two sources of data: (1) incorporating all three angles through orientation estimates from the Madgwick filter and (2) limiting ourselves to just pitch and roll derived from accelerometer-only orientation estimates. This allowed us to validate our method by comparing full 3D angular velocity against a simplified, accelerometer-limited version (Figure 5).

We computed the correlation and pairwise mean absolute difference (MAD) between the Madgwick and accelerometer-only time series to be 0.91 and 7.75 deg/s, respectively. The high correlation indicates that both methods capture similar temporal patterns of variability, which is important for our analyses of limb variability. However, the nontrivial MAD indicates that our accelerometer-only approach is less accurate in magnitude and should be interpreted as a coarse estimate rather than a precise measurement of angular velocity (Figure S1).

### 2.2. Derivable Measures

Pitch, roll, and yaw can be used to quantify the variability of orientation in the following manner. For each angle, we computed the circular standard deviation (SD) to represent individual axis variability (because pitch, roll, and yaw are angular (circular) variables, our measures were computed using circular-statistics methods). To quantify multi-axis variability, we calculated the circular covariance matrix using the pitch, roll, and yaw time series over a given time window [35]. This matrix captures the variability of each rotational axis while accounting for the circular nature of angular data. We then computed the determinant of this matrix to obtain a single scalar value of overall orientation variability. For simplicity, we will refer to this value as the “generalized variance”.

## 3. Materials and Methods

### 3.1. Ethics Statement

Our study protocol and data collection methodology were approved by the University of California, Los Angeles (UCLA) Institutional Review Board. Due to the age of the participant population and/or diagnoses that affect cognitive abilities, a legally authorized representative of all participants provided written informed consent for their data to be used in related research.

### 3.2. Participants

Study participants included typically developing infants (with a low likelihood of autism; *n* = 24) and infants with an older sibling diagnosed with autism (increased likelihood of autism; *n* = 38) enrolled in the iMove: Infant Movement Study (IRB #19-000863) at the University of California, Los Angeles.

#### 3.2.1. Increased Likelihood of Autism (ILA) Group

Inclusion criteria included status as a younger sibling of a child diagnosed with Autism Spectrum Disorder (ASD), with older sibling diagnosis confirmed by parental report and using the Social Communication Questionnaire [36]. Exclusion criteria included birth prior to 32 weeks of gestation, major intracranial abnormality, and a known genetic disorder.

#### 3.2.2. Low Likelihood of Autism (LLA) Group

Inclusion criteria included status as a younger sibling of a child with typical development. Low likelihood group exclusion criteria included birth before 36 weeks of gestation; developmental, learning, or medical conditions in any older sibling; and ASD or other neurodevelopmental disorders in any first-, second-, or third-degree relative.

### 3.3. Developmental Assessment

Developmental level of participants was evaluated using the Mullen Scales of Early Learning [37]. The Mullen Scales of Early Learning (MSEL) is a widely used developmental assessment that provides information on several subscales, including (1) fine motor, (2) visual reception, (3) receptive language, (4) expressive language, and (5) gross motor scales. Here, we utilize the gross motor subscale raw score for additional comparisons to the sensor data. The MSEL was administered by trained clinical psychologists from the UCLA Center for Autism Research and Treatment.

### 3.4. Sensor Data

APDM (Ambulatory Parkinson’s Disease Monitoring) Opal wearable sensors were used in this study; they comprise a 3D accelerometer, 3D gyroscope, and 3D magnetometer. Opal sensors are wireless and lightweight and have been used to study infant movement [12,16,38,39]. The sensor acceleration range is 6 g, and measurements are reported with 14-bit resolution. Recordings were made at 20 Hz. The data from both left and right sensors were actively synchronized throughout the recording, stored in the internal memory of each individual sensor, and downloaded at the end of each visit. The data were recorded continuously.

### 3.5. Data Collection

Leg movement data was collected from ILA and LLA infants at 3, 6, 9, and 12 months of age using the Opal wearable sensors. The protocol included visiting the infants each morning in their home environment with the goal of collecting a full day of movement data. The wearable sensors were placed on the infants’ ankles and were attached using legwarmers. Each ankle legwarmer contains a pocket to hold the wearable sensor in place. Families were instructed to go about their typical daily activities. Full-day movement data for each infant includes a standardized 5 min video segment where infants are wearing sensors and moving freely in their home environment without any caregiver-mediated movement. All analyses in this study were performed on the standardized 5 min segment.

### 3.6. Preprocessing

Since our analyses utilize gyroscope data, we first characterized and corrected for gyroscope drift. Gyroscopes are known to be susceptible to drift, which causes orientation errors to accumulate over time. To quantify this effect, we placed our sensors on a level surface in a quiet area for 30 min. We then selected a 5 min window and integrated the gyroscope readings to reveal an average offset of approximately 6 degrees. To address this, we use the imufusion library’s bias estimator, which detects quiescent periods and applies a low-pass (exponential moving average) update to the gyroscope signal to estimate and subtract a constant bias [40]. Additionally, the Madgwick filter further reduces gyroscope bias by fusing accelerometer and magnetometer data. With these methods, our angles remain stable within ±0.1 degrees over the 30 min recording duration.

To reduce high-frequency background noise and isolate the gravitational portion of accelerometer data, we chose to apply a low-pass Butterworth filter with a cutoff frequency of 8 Hz. This value was selected after conducting sensitivity analyses on a wide range of frequencies from 5 to 10 Hz and observing that most infant-induced accelerometer fluctuations occur below 8 Hz (Figure S2). Prior infant research has also used an 8 Hz cutoff frequency when evaluating infant-generated movements [41]. Additional work indicates that this cutoff preserves changes in gravitational acceleration caused by spontaneous infant movement [42,43]. Residual errors in accelerometer and magnetometer measurements, including those from transient linear acceleration and magnetic distortions, are further attenuated by the Madgwick fusion algorithm.

### 3.7. Statistical Methods

Our orientation variability measures, circular standard deviation (SD) of pitch, roll, and yaw, and generalized variance, were computed over each 5 min sample for all infants at 3, 6, 9, and 12 months in our combined infant group. To characterize the changes in infant development over time, we fit a linear mixed-effects model to examine the effect of age on each measure. Models included a cohort (ILA v. LLA) fixed effect and a linear age effect, the interaction of these terms, and random child effects to account for repeated measurements. The main effect of age was used to evaluate change over time in each variability measure. We also examined whether there were level and slope differences between ILA and LLA infants for each measure using the cohort main effect and the cohort by age interaction effect (See Appendix A.3). A separate linear mixed model combined the pitch, roll, and yaw variability data to evaluate level and slope differences between angles. This model included angle (roll, yaw v. pitch) fixed effects, a linear age effect, and their interaction, along with random child effects. Finally, at each time point, we assessed the Spearman correlation between each infant’s generalized variance and their concurrent and future MSEL gross motor (GM) scores in our combined infant group. We also conducted Pearson correlations, which are provided in the Supplementary Materials (Table S1). To maintain consistent global body posture across time points, we restricted our dataset to only non-walking infants at each visit (Table S2). Statistical significance was defined as a *p*-value less than 0.05. All analyses were performed using Python 3.9.

## 4. Results

At each time point, we indicate the sample sizes of unique participants stratified into the ILA and LLA cohorts (Table 1).

For each age, we report the group mean ± SE (standard error) of our variability measures across infants in the combined infant group (Table 2). Estimated means for each measure are also provided separately for ILA infants (Table A1) and LLA infants (Table A2) (see Appendix A.3).

Linear mixed-effects models showed that the circular standard deviations of pitch, roll, and yaw increased significantly over time in our combined infant sample (Table 3). Roll and yaw circular SD displayed greater increases per month (3.63° ± 0.52, 4.17° ± 0.52, both with *p* < 0.001) compared to pitch circular SD (0.71° ± 0.28, *p* = 0.01) [Table 3]. Generalized variance also exhibited a significant increase over time (0.06 ± 0.01, *p* < 0.001), indicating greater overall variability in 3D orientation over time.

Regression analyses showed that both roll and yaw circular SDs were significantly larger than pitch on average across the 3, 6, 9, and 12 month time points (Table 4). Our analyses also identified significant differences in slope for both roll vs. pitch and yaw vs. pitch, indicating that the circular SDs of roll and yaw increased more rapidly with age compared to pitch (Table 5).

We generally did not observe significant differences between ILA and LLA infants in either level or slope (see Appendix A.3).

We found positive correlations between generalized variances and MSEL GM scores among our infant sample (Table 6). In particular, generalized variance of 9 month infants showed significantly positive values with 9 month and 12 month MSEL GM Scores (r = 0.51, r = 0.55).

## 5. Discussion

Here, we developed and applied a processing pipeline for viewing infant movement that relies on gravitational acceleration to compute the metrics pitch, roll, and yaw. Gravitational acceleration consists of a large and rich portion of accelerometer data and can be used in addition to measures of movement-related acceleration to improve our understanding of typical and atypical infant motor development. By describing 3D infant limb orientation with pitch, roll, and yaw, we can capture additional aspects of movement, such as coordination and postural repertoire, that are not represented by traditional linear kinematic measures. Infant limb orientation patterns are often impacted in neuromotor conditions such as cerebral palsy where spasticity and hypotonia can impact the ability to achieve typical locomotor patterns. Rehabilitation strategies for cerebral palsy often aim to improve the ability to achieve functional movement [44]. Developing objective wearable-sensor metrics to quantify orientation could therefore support tracking longitudinal changes in response to intervention, both in the clinic and in the home. Video-based work has also shown that infant postural patterns, such as body asymmetries and postural stability, can be informative for later neuromotor outcomes, including cerebral palsy, by implicitly encoding limb orientation [32]. Complementing video- and observation-based approaches, wearable sensors have leveraged inertial signals that also inherently reflect limb orientation to classify infant postures and movements across development (e.g., sit-to-stand transitions and falls) [45]. Given the clinical relevance of limb orientation, pitch, roll, and yaw offer an explicit and quantifiable representation of segment orientation, which allows for derivation of many important aspects of infant postural control and locomotive changes.

Our team has also developed measures of movement variability to classify infant movement in the first year of life and examine how differences in movement variability manifest in infants with a higher likelihood of developing neurodevelopmental disorders. Movement variability is a hallmark of healthy neuromotor development and is theorized to support the development and execution of later fundamental motor skills [46,47]. Because many developing motor skills depend on how infants orient their limbs and body relative to gravity, variability in limb orientation is likely an important component of infant movement variability and typical motor development. Thus, we applied our method to measure variability in infant limb orientation. As a preliminary analysis, we summarized limb orientation variability from pitch, roll, and yaw using the circular standard deviation (axis-specific variability) and the generalized variance (overall multiaxis variability). We then analyzed age-related changes in our measures and whether these orientation dynamics relate to standardized gross motor outcomes.

In our longitudinal data, we observed significant increases in orientation variability measures over time. These increases in variability from 3 to 12 months of age may reflect expected developmental changes in posture and orientation transitions during the first year of life when infants rapidly develop motor skills [48]. Early in infancy, most movements are spontaneously generated leg and arm movements that occur while infants are supine [49]. In the first year of life, infants learn locomotor behaviors such as sitting, crawling, and walking to explore their environment, which increase orientation transitions and their overall repertoire of orientations [50]. Additionally, because roll and yaw exhibited greater level and slope differences in variability than pitch, future metrics and analyses may consider weighting these angles more heavily when tracking developmental change. Together, pitch, roll, and yaw derived from wearable sensors offer a practical approach to quantify important age-related changes across the first year of life, enabling remote monitoring of these orientation dynamics in naturalistic, home settings.

We also examined the association of our orientation measures with the Mullen Scales of Early Learning (MSEL) gross motor subscale, which is a widely used standardized measure of infant motor function. At 3 and 6 months, the generalized variance showed relatively weak associations with MSEL gross motor items (Table 6). One likely explanation of the weaker correlations at these ages is that there is a mismatch in what the MSEL captures compared to the sensor-based variance measure. For instance, early MSEL gross motor items at 3 months of age emphasize head and trunk control (e.g., “Rotates head” and “Held upright, holds head steady”), whereas our variability measures primarily monitor leg orientation dynamics [37]. At 9 months, generalized variance displayed significant positive correlations with MSEL gross motor scores (Table 6). As infants approach this age, they begin to develop gross motor skills that require larger and more frequent changes in limb orientation in preparation for being ambulatory (e.g., “Rolls over” and “Shifts weight, reaches”), which may better align with what our measures capture. Notably, the significant correlation between 9-month generalized variance and later 12-month MSEL gross motor score indicates that orientation variability may have utility for predicting later gross motor development. Our preliminary analyses suggest that measures derived from pitch, roll, and yaw show correlation to a standardized motor measure which strengthens the clinical and research utility of this approach to examine infant motor function. Examining the association of these variability metrics to other common standardized measures of infant gross motor function will help inform the association of these quantitative metrics to motor function in earlier and later infant ages.

Although our metrics have proven useful for tracking infant limb orientation during 5 min segments, the limitations of this method should be noted. Circular standard deviation and the generalized variance provide a coarse summary of orientation variability within a given segment, but they do not capture the temporal structure of how these angles change over time. Incorporating time-sensitive features, and nonlinear analyses such as recurrence quantification analysis (RQA), can capture the sequencing and repetition within a segment that our current measures do not capture [51]. These measures also rely on concurrent videos to provide behavioral context for pitch, roll, and yaw, which faces scalability challenges for larger, full-day recordings. Video available for a subset of segments can be used to provide ground-truth behavioral labels for training context-detection machine learning models, which can then be applied to longer sensor recordings without video. Finally, because this study was conducted with a modest sample size, its statistical power and generalizability may be limited. These findings will benefit from validation in a larger cohort, which we aim to address in future studies.

In conclusion, this manuscript introduces a complementary method of characterizing infant movement by using the gravitational domain of accelerometer data to quantify infant limb orientation dynamics. Building on well-established gravity-referenced orientation estimation methods, our work contributes a novel perspective to the literature on infant development, which has traditionally emphasized linear movements. Future analyses that couple orientation variability with existing kinematic measures have the potential to improve sensitivity to atypical motor outcomes in populations, such as infants that are classified as being at higher likelihood of neurodevelopmental disorders. This includes infants with perinatal brain injury, prematurity, low birth weight, family history of autism, and those with genetic conditions. Additionally, future work will extend our orientation perspective by integrating conceptually derived measures that leverage the temporal structure of our high-frequency data, such as complexity, curvature, and entropy, with data-driven feature discovery through machine learning approaches. More broadly, the framework we propose can be applied to other infant groups at risk of motor delays such as cerebral palsy, where posture and orientation may provide early indicators and intervention targets. Consideration of these approaches can add to the field’s ability to utilize wearable sensors to capture important aspects of infant motor development.

## Figures and Tables

**Table 1:** ILA and LLA infant sample sizes at each time point (3, 6, 9, and 12 months).

	3 Month	6 Month	9 Month	12 Month
**ILA infants**	25	29	31	30
**LLA infants**	17	17	17	19
**Total**	42	46	48	49

**Table 2:** Mean ± SE circular SD of pitch, roll, and yaw in degrees, and generalized variance across ages in the combined infant group.

	3 Month	6 Month	9 Month	12 Month
**Pitch Circular SD**	14.8 ± 4.9	20.3 ± 7.1	18.6 ± 9.3	23.4 ± 8.8
**Roll Circular SD**	21.4 ± 11.7	34.4 ± 16.0	44.7 ± 17.5	52.7 ± 13.8
**Yaw Circular SD**	24.3 ± 12.8	32.2 ± 14.0	50.4 ± 19.4	59.2 ± 13.5
**Generalized Variance**	0.025 ± 0.115	0.093 ± 0.284	0.303 ± 0.469	0.491 ± 0.568

**Table 3:** Estimated age-related slopes for circular SD of pitch, roll, and yaw and generalized variance in the combined infant group.

	Estimated Slope (Per Month)	*p*-Value
**Pitch Circular SD**	0.71 ± 0.28	0.010
**Roll Circular SD**	3.63 ± 0.52	<0.001
**Yaw Circular SD**	4.17 ± 0.52	<0.001
**Generalized Variance**	0.06 ± 0.01	<0.001

**Table 4:** Average level difference in circular SD for roll vs. pitch and yaw vs. pitch across combined infant group.

	Estimated Difference (Degrees)	*p*-Value
**Roll v. Pitch Circular SD**	19.1 ± 1.2	<0.001
**Yaw v. Pitch Circular SD**	22.2 ± 1.2	<0.001

**Table 5:** Average slope difference in circular SD for roll vs. pitch and yaw vs. pitch across combined infant group.

	Estimated Difference (Degrees/Month)	*p*-Value
**Roll v. Pitch Circular SD**	2.64 ± 0.37	<0.001
**Yaw v. Pitch Circular SD**	3.28 ± 0.37	<0.001

**Table 6:** Correlations between generalized variance and MSEL gross motor (GM) scores (non-walking infants).

	3 M Generalized Variance	6 M Generalized Variance	9 M Generalized Variance	12 M Generalized Variance
**3 M MSEL GM**	r = 0.01			
**6 M MSEL GM**	r = −0.03	r = 0.09		
**9 M MSEL GM**	r = −0.01	r = 0.22	r = 0.51 *	
**12 M MSEL GM**	r = 0.12	r = 0.34	r = 0.55 **	r = 0.15

* *p* < 0.01, ** *p* < 0.001.

## Data Availability

The data presented in this study are available on request from the corresponding author.

## Supplementary Materials

The following supporting information can be downloaded at: https://www.mdpi.com/article/10.3390/s26072274/s1, Figure S1: Bland–Altman plot comparing composite angular velocity estimated from accelerometer-only data and from the Madgwick filter, showing reasonable agreement between the two methods; Figure S2: Comparison of left leg tri-axial accelerometer data after applying 5 Hz and 8 Hz low-pass Butterworth filters. No clear visual differences were observed between the filtered signals; Table S1: Pearson correlations between generalized variance and MSEL gross motor (GM) scores (non-walking infants); Table S2: Sample sizes used for correlation analyses between generalized variance and MSEL gross motor scores at each time point.

## Appendix A

In this section, we provide implementation details and additional supporting analyses. Appendix A.1 explains why the yaw angle cannot be calculated from accelerometer data alone without additional gyroscope and magnetometer information. Appendix A.2 describes the steps taken to convert pitch, roll, and yaw time series into a composite angular velocity signal using quaternion angular distance and smoothing. Appendix A.3 reports descriptive longitudinal summaries of our orientation-variability measures stratified by risk group (Table A1 and Table A2), including circular standard deviations of pitch, roll, yaw, and tilt as well as generalized variance.

### Appendix A.1

Since our current method consists only of accelerometer data, it cannot capture rotations of the sensor about the normal vector itself (i.e., Earth’s *z*-axis) without additional gyroscope and magnetometer data. Thus, a notable limitation arises when the infant produces a movement purely about the Earth’s vertical axis between frames. Such motion does not alter the accelerometer readings and is therefore undetectable in our current setup.

### Appendix A.2

To translate pitch and roll into an angular velocity time series, we performed the following steps at each frame. When only pitch and roll were available, we used them to construct individual Euler rotation matrices representing rotations about the *y*-axis and *x*-axis, respectively. These matrices were then multiplied together to form a composite matrix. This matrix was converted into a quaternion, partially representing the orientation of the surface normal vector.

When yaw data was available, we constructed an additional Euler rotation matrix about the *z*-axis and incorporated it into our composite matrix through matrix multiplication. This yielded a full orientation quaternion that accounted for rotation about all three axes.

For each quaternion time series, we calculated the standard quaternion angular distance between successive quaternions with the formula $\theta =2arccos(|q_{1}\cdot q_{2}|)$ to represent the change in orientation from frame to frame. Because our data was recorded at constant time intervals, this angular distance can be interpreted as the rate of orientation change at each time point providing a time series of angular velocities. Finally, to reduce background noise, we applied a 1D Gaussian smoothing filter (window length: 5) to our angular velocity time series.

### Appendix A.3

**Table A1:** Mean ± SE circular SD of pitch, roll, and yaw in degrees, and generalized variance across ages in the ILA infant group.

	3 Month	6 Month	9 Month	12 Month
**Pitch Circular SD**	14.5 ± 5.0	21.1 ± 7.8	20.2 ± 8.6	23.5 ± 9.1
**Roll Circular SD**	21.3 ± 12.1	36.5 ± 18.1	44.9 ± 16.8	52.1 ± 15.1
**Yaw Circular SD**	23.1 ± 11.7	36.1 ± 16.1	52.4 ± 18.6	58.0 ± 15.8
**Generalized Variance**	0.033 ± 0.149	0.139 ± 0.351	0.313 ± 0.422	0.499 ± 0.538

**Table A2:** Mean ± SE circular SD of pitch, roll, and yaw in degrees, and generalized variance across ages in the LLA infant group.

	3 Month	6 Month	9 Month	12 Month
**Pitch Circular SD**	15.2 ± 4.8	19.1 ± 5.5	15.7 ± 9.7	23.1 ± 8.4
**Roll Circular SD**	21.5 ± 11.0	31.0 ± 11.0	44.3 ± 18.6	53.7 ± 11.4
**Yaw Circular SD**	26.0 ± 14.2	25.7 ± 5.1	46.8 ± 20.4	61.1 ± 8.4
**Generalized Variance**	0.014 ± 0.024	0.013 ± 0.017	0.284 ± 0.558	0.477 ± 0.628
