# Supplementary material for: A Novel Workflow to Estimate Limb Orientation from Wearable Sensors to Monitor Infant Motor Development
Source: Sensors (Basel). 2026 Apr 7;26(7):2274. doi: 10.3390/s26072274 (PMC13075267; doi:10.3390/s26072274)
Supplement: Supplementary file 1 [file sensors-26-02274-s001.zip › sensors-4184712-supplementary.pdf]

Supplementary Materials

Supplementary Figure S1: Bland–Altman plot comparing composite angular velocity estimated from accelerometer-only data and from the Madgwick filter, showing reasonable agreement between the two methods.

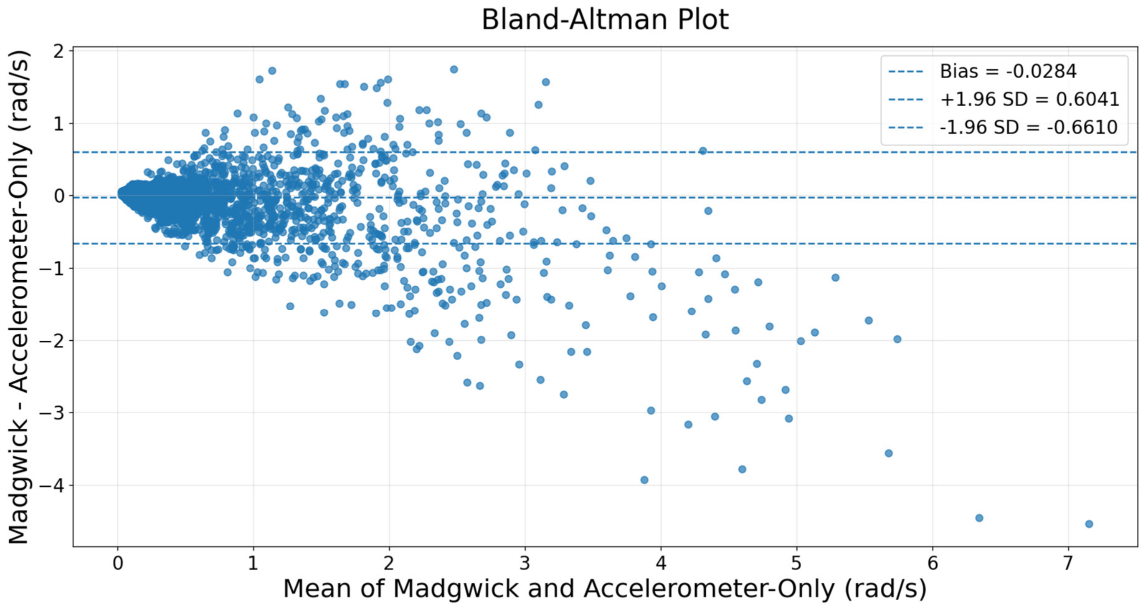

Supplementary Figure S2: Comparison of left leg tri-axial accelerometer data after applying 5 Hz and 8 Hz low-pass Butterworth filters. No clear visual differences were observed between the filtered signals.

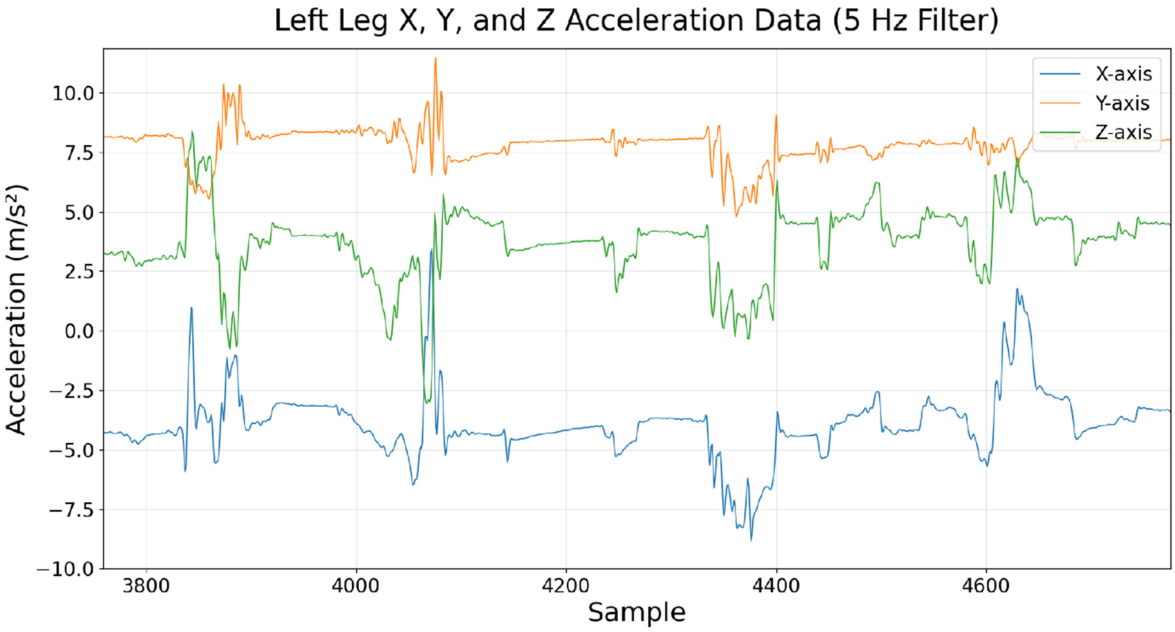

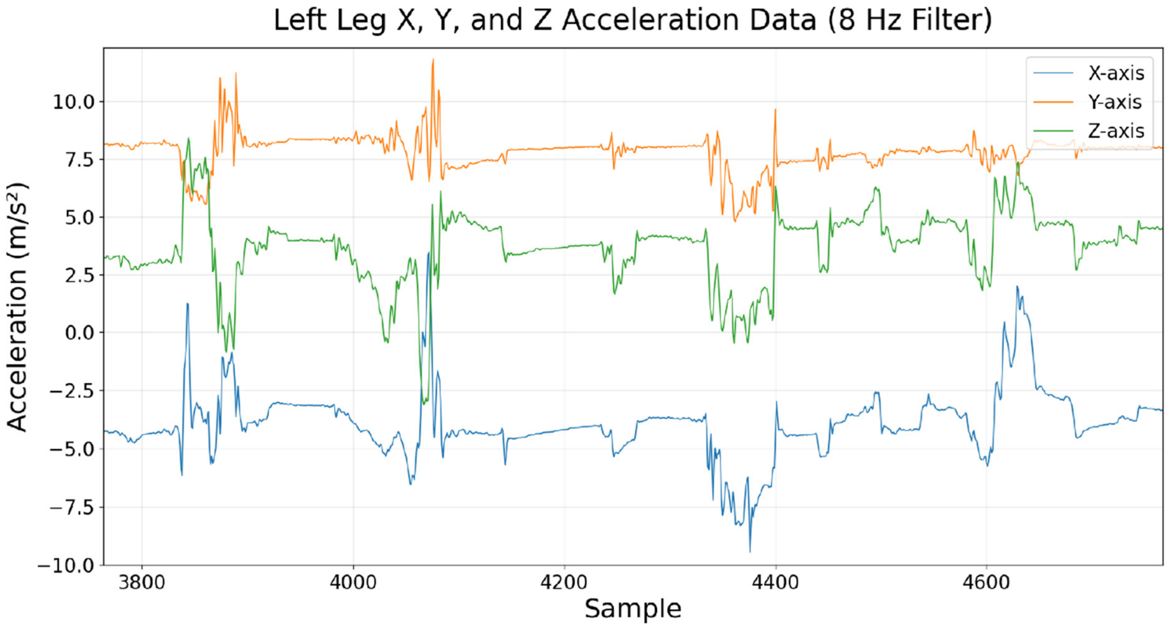

Supplementary Table S1: Pearson correlations between generalized variance and MSEL gross motor (GM) scores (non-walking infants)

	**3M Generalized Variance**	**6M Generalized Variance**	**9M Generalized Variance**	**12M Generalized Variance**
**3M MSEL GM**	r = 0.29			
**6M MSEL GM**	r = 0.17	r = 0.09		
**9M MSEL GM**	r = 0.11	r = 0.27	**r = 0.51***	
**12M MSEL GM**	r = 0.14	r = 0.16	**r = 0.64****	r = 0.30

* $p < 0.01$, ** $p < 0.001$.

Supplementary Table S2: Sample sizes used for correlation analyses between generalized variance and MSEL gross motor scores at each time point.

	**3M Generalized Variance**	**6M Generalized Variance**	**9M Generalized Variance**	**12M Generalized Variance**
**3M MSEL GM**	N = 30			
**6M MSEL GM**	N = 29	N = 31		
**9M MSEL GM**	N = 24	N = 27	N = 31	
**12M MSEL GM**	N = 21	N = 29	N = 34	N = 19
